# Effects of soil properties and microbial community composition on ginsenosides accumulation in farmland ginseng

**DOI:** 10.3389/fbioe.2024.1462342

**Published:** 2024-09-06

**Authors:** Tao Zhang, Zhefeng Xu, Yibing Wang, Qiao Gao

**Affiliations:** ^1^ School of Pharmacy, Changchun University of Traditional Chinese Medicine, Changchun, China; ^2^ Changchun University of Traditional Chinese Medicine, Jilin Provincial Institute of Ginseng Science, Changchun, China

**Keywords:** Panax ginseng, ginsenosides, soil physicochemical properties, soil enzyme activity, soil microbial communities

## Abstract

Soil is the material basis of ginseng survival, and revealing the correlation between soil and ginsenoside has far-reaching significance for the sustainable development of ginseng industry. In this study, the content of ginsenosides in 3-year-old ginseng roots, the physicochemical properties of rhizosphere soil and the microbial community composition were studied. The results showed that the contents of total saponins in different months were significantly different. The richness and diversity of soil microbial community decreased with the extension of planting time. The activities of complexed iron, organic matter and protease in soil had significant effects on α-diversity of soil microbial community. Functional gene analysis showed that carbon sequestration, protein translation, nitrogen metabolism, transcription factors and chlorophyll metabolism were the main functions of soil bacterial community. The results of correlation analysis and redundancy analysis showed that *pH*, available potassium, organic matter, complexed iron, Firmicutes and Acidobacteria were the key factors affecting ginsenoside accumulation. The changes of soil physical and chemical properties affect the abundance of soil microorganism, and the accumulation of ginsenoside in ginseng is affected by soil microorganism. A co-regulatory network of Physicochemical Properties-Microbe-Ginsenoside was established. To provide theoretical support for the cultivation of ginseng.

## 1 Introduction

*Panax ginseng* C. A. Meyer is a perennial plant of the genus Panax in the family Araliaceae, which is one of the three treasures of northeastern China. With a long history and characteristics of traditional Chinese medicine, but also has a very rich nutritional value and the most important medicinal activity of Chinese medicine (China, 2020). Ginseng is mainly distributed in 33–48°N, including China, North Korea, South Korea, Russia and Japan. Due to the destruction of the ecological environment of cultivated ginseng, people’s long-term overexploitation has led to fewer and fewer wild resources, and cultivation has become the main source of ginseng (Li et al., 2019). The main mode of ginseng cultivation is to cut down forests to cultivate ginseng, which will lead to serious damage to forests and is not conducive to the sustainable development of traditional Chinese medicine resources (Weihao et al., 2016). Studies have shown that the quality of ginseng cultivated in farmland can meet the standard requirements of the national pharmacopoeia, and the content of ginsenosides in its pharmacodynamic component is even higher than that of ginseng cultivated by logging (Baowen et al., 2016), so it is of great significance to develop ginseng cultivated in farmland.

Soil is closely related to medicinal plants, providing them with the water and nutrients they need to grow and develop, thus ensuring that they can complete their metabolism and produce and accumulate the nutrients they need. Soil moisture, pH, available nutrients, trace elements and enzyme activities can directly or indirectly affect the growth and development of medicinal plants, and even affect the content of their active ingredients (Applequist et al., 2020; Xu et al., 2021). Soil microorganisms play a very important role in the soil ecosystem, which has the functions of maintaining soil microecological balance, promoting the decomposition and transformation of organic matter, participating in mineral nutrient cycle, and promoting the formation and improvement of soil structure. Soil microorganisms are sensitive to changes in the soil environment, and together with enzymes in soil, they are called the most sensitive activity indicators of soil. The diversity and richness of its community structure can reflect the health of the soil. In general, healthy soils have a large number of beneficial microorganisms and a relatively small number of pathogenic bacteria (Bever et al., 2015; Xiao et al., 2020). The rhizosphere zone of plants is a soil microzone where plant roots communicate with the soil microenvironment actively, and it is a special microecological area for the interaction between plant roots, soil and microorganisms. The flow of energy and the conversion of matter in the rhizosphere region are extremely strong, so it can be used as an important indicator to study the changes of soil microecology, and the relationship between plants and soil in the rhizosphere can better clarify the changes between plant roots and soil microenvironment (Bennett et al., 2017; Semchenko et al., 2018; Bennett and Klironomos, 2019).

Ginseng grows slowly and takes 5–6 years before it can be used clinically. The cultivation time is an important factor affecting the quality of ginseng, and ginsenosides are the main pharmacodynamic components of ginseng and a key biomarker to evaluate the medicinal quality of ginseng (Liang et al., 2019). The content of ginsenosides in ginseng increases with the number of years of cultivation. As the cultivation years of ginseng increase, the organic matter of the soil is consumed a lot, and the nutrients including nitrogen, phosphorus, and potassium are significantly reduced, so the fertility of the soil decreases (Zhang et al., 2014; Yin et al., 2022). Soil acidity changed significantly with the increase of planting years, and the contents of organic matter, nitrate nitrogen and available phosphorus decreased significantly (Xi et al., 2022). At present, there are few studies on the correlation between ginsenoside content and soil factors and soil microbial communities during ginseng cultivation, and the mechanism of the influence of soil factors and soil microorganisms on ginsenoside accumulation is still unclear. Therefore, it is of far-reaching significance to study the correlation between the quality of farmland ginseng and soil factors for the cultivation of high-quality ginseng and the sustainable development of the ginseng industry. In this study, the contents of 11 monomeric saponins and total saponins in different months of 3-year-old ginseng were determined, and the physical and chemical properties, enzyme activities and soil microbial community composition of rhizosphere soil were detected, and the soil factors and microbial factors affecting the content of ginsenosides were explored through correlation analysis and redundancy analysis, so as to provide a theoretical and practical basis for the scientific cultivation of ginseng and the improvement of the medicinal efficacy of ginseng.

## 2 Materials and methods

### 2.1 Plant material

The test material was collected in Fusong Town, Fusong County, Baishan City, Jilin Province (127°11′, E 42°38′N). The place belongs to the cold temperate humid climate zone, the average annual temperature is 4°C, the altitude is 750 m, the four seasons are distinct, the average annual rainfall is 800 mm, the winter is long, cold, and the snow is deep; Summers are short, hot, and rainfall is concentrated. On 20 April 2022, ginseng transplanting was carried out, and 2-year-old healthy ginseng seedlings of similar size were transplanted to the farmland. Ginseng samples and soil samples were collected on the 20th of each month from June to October 2022. Ginseng samples were collected by five-point sampling method, and 10–20 ginseng samples were collected from each test site. The whole root system of ginseng was completely excavated, the rhizosphere soil (≤5 cm away from the taproot) was collected, weeds, stones and plant residues in the soil were removed, mixed and divided into two parts and packed into sterile ziplock bags. One aliquot was frozen in liquid nitrogen and stored in a −80°C freezer after being brought back to the laboratory for the determination of soil microorganisms; The other copy was kept in dry ice and brought back to the laboratory in a 4°C freezer for soil physicochemical properties and enzyme activity analysis. The ginseng samples with small individual differences and no pests and diseases were screened and washed with deionized water to remove sediment, and the surface moisture was absorbed with filter paper and dried naturally to constant weight for ginsenoside content analysis.

### 2.2 Analysis of ginsenosides by HPLC

Milled powder from heat-dried roots was separated using a 60-mesh sieve, and 1.0 g milled powder was weighed once from each of three technical replicates. The determination of the content of 11 monomeric ginsenosides (Rg1, Re, Ro, Rb1, Rb2, Rb3, Rf, Rc, Rd, Rg3, Rh2) was determined by Zhang T et al. (2021), using a Thermo Ultimate 3000 HPLC, the chromatographic column was Elite Hypersil ODS2 (250 mm × 4.6 mm, 5 μm), the mobile phase was acetonitrile (A)-water (B), gradient elution (0–17 min, 12% ∼18% A; 17∼29 min, 19%∼27%A; 30∼31 min, 27%∼31%A; 32∼52 min, 31%∼35%A; 52∼69 min, 35%∼80%A; 69–85 min, 80%–12%A), the flow rate was 1.0 mL/min, the column temperature was 25°C, the detection wavelength was 203 nm, and the injection volume was 10 μL. After extracting ginsenosides from ginseng using a methanol ultrasonic extraction method, the content of ginseng total saponins (TG) was determined by a method of measuring the absorbance value at 544 nm after vanillin-concentrated sulfuric acid color development (Zhang et al., 2019).

### 2.3 Analysis of soil properties

The *pH* meter measures the pH of the soil (the soil-water ratio is 1:5). The conductivity meter was used to determine the electrical conductivity (EC) (the soil-water ratio was 1:5). The bulk density (BD) of the soil was determined by the ring knife method. Soil water content (SWC) was determined by drying method. Alkali-hydrolyzable nitrogen (AN) was determined by alkaline hydrolysis diffusion method. Available phosphorus (AP) was determined by sodium bicarbonate extraction-molybdenum blue colorimetric method. Available potassium (AK) is determined by flame photometry. Potassium dichromate oxidation-ferrous ammonium sulfate titration to determine the content of organic matter (OM). Potassium permanganate cold digestion-o-phloline colorimetric determination of complexed iron (Fe). Acetic acid buffer extraction-silicon-molybdenum blue colorimetric method for the determination of available silicon (Si). Indigophenol blue colorimetric assay was used to determine urease (Ure) activity. Potassium permanganate titration was used to determine catalase (CAT) activity. Ninhydrin colorimetric assay for protease (Prot) activity. The activities of sucrase (Inv), amylase (AMY), cellulase (Cel) and dextranase (Dex) were determined by 3,5-dinitrosalicylic acid colorimetric method. Phenol disulfonic acid colorimetric assay was used to determine nitrate reductase (NR) activity. Acid phosphatase (ACP) activity was determined by colorimetric sodium phosphate. The above soil determination methods refer to "Soil Agrochemical Analysis” and "Soil Enzymes and Their Research Methods" (Others, 1986; Shidan, 2000).

### 2.4 Sequencing of 16S rDNA and ITS genes

DNA from soil samples was extracted using a soil DNA kit (D5625, Omega, Inc., United States). The bacterial 16s V3-V4 region and fungal ITS1 gene were amplified using the 341F-805R (5′-CTACGGGNGGCWCWGCAG-3′/5′-GACTACHVGGGTATCTAATCC-3′) and ITS1FI2-ITS2 (5′-GAACCWGCGGARGGATCA-3′/5′-GCTGCGTTCTTCATCGATGC-3′) primer sets. Amplicon synthesis, library construction, and Illumina NovaSeq sequencing (2 × 250bp) were performed at Lianchuan-Biotech Co., Ltd (Hangzhou, Zhejiang Province, China).

### 2.5 Sequence processing and statistical analysis

After the sequencing is completed, the raw data RawData is obtained, and the double-ended data is spliced by overlap, and the quality control and chimera filtering are carried out to obtain high-quality CleanData. DADA2 obtains single-base-accurate representative sequences through steps such as "dereplication” (equivalent to clustering with 100% similarity), which improves data accuracy and species resolution. The core of DADA2 is denoising, and then the concept of ASVs (Amplicon Sequence Variants) is used to construct an OTUs-like (Operational Taxonomic Units) table, and the final ASV feature table and feature sequence are obtained, and further diversity analysis, species taxonomic annotation and difference analysis are carried out. The traits and classification tables with these classifications have been removed: chloroplasts, mitochondria, and other unclassified parts. Alpha diversity index, principal coordinate analysis (PCoA), and non-metric multidimensional scaling (NMDS) were performed using the phyloseq package in R (J et al., 2013), and redundancy analysis (RDA) and similarity analysis (ANOSIM) were performed using vegan packaging in R (Oksanen et al., 2020). SPSS 21.0 software was used for one-way ANOVA and Pearson correlation analysis, and the one-way variance was tested by least significance difference (LSD), and Pearson test was used for Pearson correlation analysis, and *p* < 0.05 was used to indicate that the difference was statistically significant. Functional prokaryotic Taxa annotations (FAPROTAX, http://www.zoology.ubc.ca/louca/FAPROTAX/) and FUNGGuild (https://github.com/UMNFuN/FUNGGuild) to predict the functional groups of bacteria and fungi. Plotting was performed in GraphPad Prism 8.2 and the Lianchuan Bio Cloud Platform (https://www.omicstudio.cn/tool) provided by Lianchuan Biotech.

## 3 Results

### 3.1 Ginsenoside content at different growth stages

**FIGURE 1:**
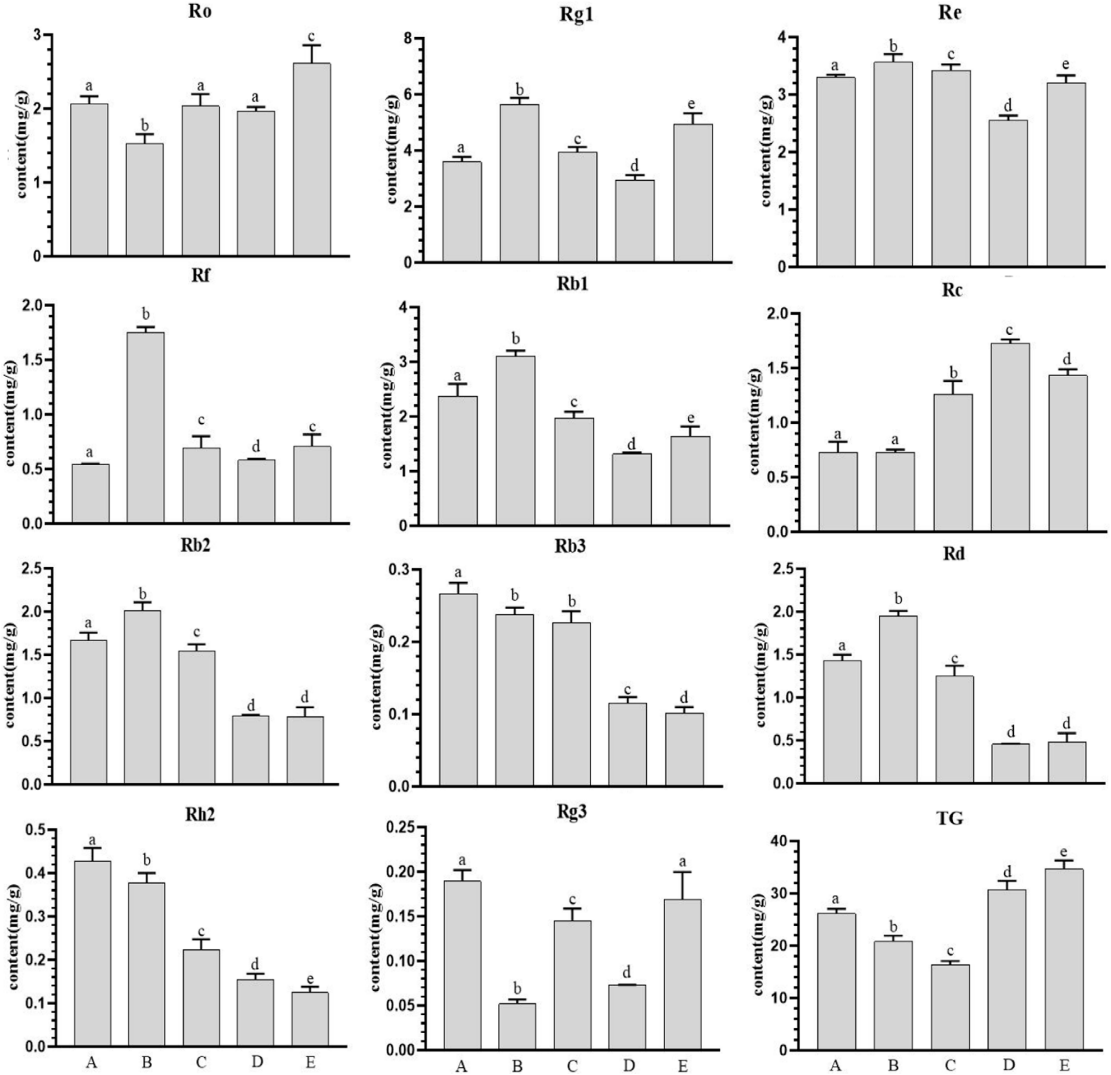
Ginsenoside content in farmland ginseng at different periods. Note: **(A)**: June 20th; **(B)**: July 20th; **(C)**: August 20th; **(D)**: September 20th; **(E)**: October 20th. Different 4 lowercase letters in the same analysis indicator indicate significant differences (*p*<0.05).

The contents of 11 ginseng monomer saponins and total saponins in ginseng at different growth stages had different trends (Figure 1), and the overall trend of ginsenosides Ro, Rg1, Rf, Rc and total saponins increased gradually during this period, and the difference reached a significant level. The remaining seven ginsenosides showed a downward trend. The maximum values of Rg1, Re, Rf, Rb1, Rb2 and Rd were found on July 20, and the maximum values of Re and total saponins were found on October 20. There was a significant difference in most types of ginsenosides with the change of month (*p* < 0.05).

### 3.2 Changes in physicochemical properties and enzyme activities of ginseng rhizosphere soil

The results showed that soil *pH*, water content, bulk density, AK, Si, Fe, Dex and NR showed a gradual decreasing trend, while AN, AP, Prot, Cel, AMY and ACP showed a gradual increasing trend (Table 1). The differences were all significant. The minimum values of soil *pH*, bulk density, water content, organic matter and AK all occurred in September, and the minimum values of Fe, Si, CAT and NR all appeared in October.

**TABLE 1:** Physicochemical properties and enzyme activity of ginseng rhizosphere soil at different periods.

Index	unit	A	B	C	D	E
*pH*	-	4.77 ± 0.02a	4.50 ± 0.04b	4.64 ± 0.03c	4.39 ± 0.05d	4.41 ± 0.04d
EC	μS/cm	163.30 ± 14.07a	199.77 ± 12.17b	128.30 ± 4.74c	187.43 ± 10.36d	144.27 ± 2.86e
BD	g/cm^3^	0.98 ± 0.02a	0.98 ± 0.01a	0.92 ± 0.05b	0.81 ± 0.03c	0.84 ± 0.02d
SWC	%	28.61 ± 0.85a	28.02 ± 0.23a	24.19 ± 0.20b	19.75 ± 1.40c	23.98 ± 0.11d
AN	mg/kg	167.42 ± 10.60a	231.25 ± 12.42b	167.49 ± 11.00a	214.60 ± 13.82c	255.52 ± 7.21d
AP	mg/kg	11.30 ± 0.42a	12.57 ± 0.42b	26.35 ± 1.25c	11.45 ± 0.48a	12.78 ± 0.40b
AK	mg/kg	139.76 ± 13.49a	53.92 ± 9.45b	20.64 ± 7.39c	17.78 ± 7.27c	24.12 ± 10.00c
OM	g/kg	87.73 ± 1.50a	88.91 ± 1.40a	85.23 ± 1.10b	82.70 ± 1.74c	100.08 ± 1.67d
Fe	mg/kg	83.28 ± 6.64a	109.37 ± 6.72b	82.75 ± 4.51a	74.75 ± 3.96c	72.60 ± 4.91c
Si	mg/kg	69.41 ± 6.61a	45.37 ± 2.04b	49.79 ± 2.93c	57.55 ± 3.43d	31.95 ± 4.05e
Ure	mg/g/d	49.66 ± 4.58a	76.81 ± 3.37b	15.41 ± 2.70c	52.86 ± 6.88a	70.74 ± 4.05d
CAT	mg/g/20min	2.54 ± 0.11a	2.48 ± 0.10a	2.67 ± 0.05b	2.55 ± 0.08 ab	2.40 ± 0.05c
Prot	μg/g/d	53.64 ± 5.35a	107.87 ± 6.05b	211.19 ± 4.05c	270.37 ± 11.93d	173.74 ± 12.38e
Inv	mg/g/d	21.36 ± 2.43a	21.20 ± 2.56a	17.63 ± 1.05b	31.56 ± 2.30c	19.87 ± 2.14 ab
AMY	mg/g/d	1.19 ± 0.31a	1.77 ± 0.36a	2.40 ± 0.21b	2.59 ± 0.23b	6.06 ± 1.78c
Cel	mg/g/d	0.12 ± 0.02a	0.41 ± 0.10b	2.15 ± 0.33c	0.39 ± 0.14b	0.14 ± 0.02a
Dex	mg/g/d	0.19 ± 0.05a	0.10 ± 0.02b	0.32 ± 0.03c	0.15 ± 0.04 ab	0.13 ± 0.02 ab
NR	μg/g/d	48.93 ± 3.14a	46.11 ± 2.53 ab	45.06 ± 1.21b	20.97 ± 2.11c	3.67 ± 0.51d
ACP	mg/g/d	5.38 ± 1.72a	7.59 ± 0.64b	7.73 ± 0.73bc	8.17 ± 0.93c	6.11 ± 0.49a

Note: A: June 20th; B: July 20th; C: August 20th; D: September 20th; E: October 20th. Different lowercase letters in the same analysis indicator indicate significant differences (p<0.05).

### 3.3 Richness and diversity of soil bacterial and fungal communities

**FIGURE 2:**
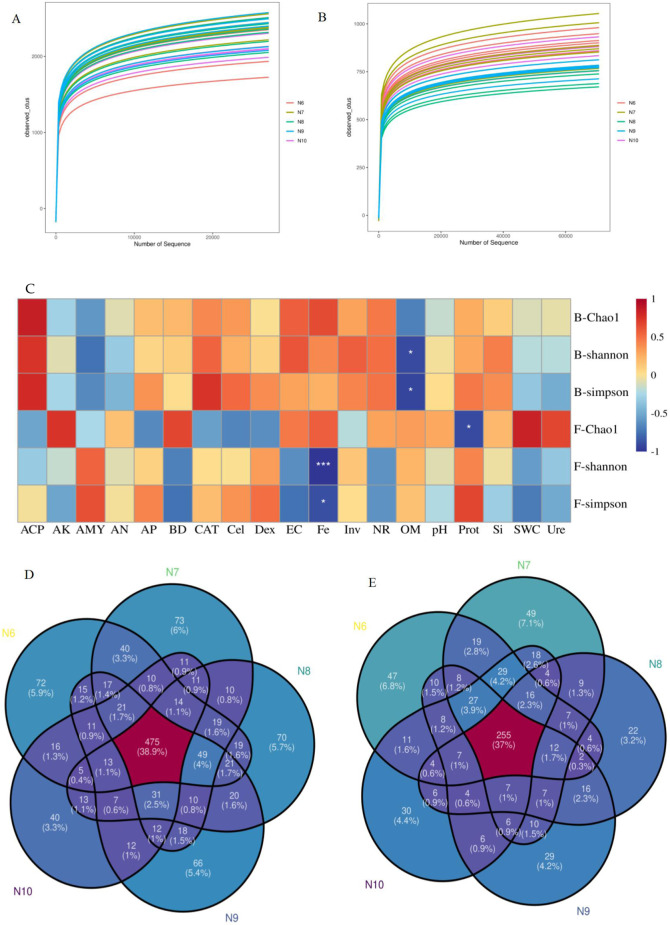
Analysis of soil microorganisms. **(A, B)** Dilution curves of soil bacteria (2**A**) and fungi (2**B**) in the rhizosphere of ginseng at different stages; **(C)**. Pearson correlation analysis of α diversity index of soil bacterial and fungal communities with soil factors (*:*p*<0.05; **:*p*<0.01; ***:*p*<0.001), Note: B- represents the bacterial community and F- represents the fungal community. **(D, E)** Venn plot of the number of OTUs in bacterial (2**D**) and fungal (2**E**) diversity

In all samples, 1,468,619 bacterial and 2,324,943 fungal effective sequences were obtained after eliminating the bias caused by different sequencing depths. Based on the similarity level of 97%, a total of 12,438 bacterial OTUs and 4,195 fungal OTUs were obtained, and the dilution curves of bacteria (Figure 2A) and fungi (Figure 2B) slowed down with the increase of sequencing depth, indicating that the number of bacteria and fungi tended to flatten with the increase of sequence number, indicating that the amount of sequencing data was reasonable. The coverage of OTUs in each sample was higher than 98% (Table 2), indicating that the sequencing results were well representative, covering the biological information of most of the bacteria and fungi in the sample. The representative sequences were amplified in the RDP database, and the bacterial sequencing data were clustered into 1,221 genera and 50 phyla, and the fungal sequencing data were clustered into 689 genera and 15 phyla.

**TABLE 2:** Bacterial and fungal community diversity indices of ginseng rhizosphere at different stages.

Number	Bacterium					Fungus				
	observed_OTUs	Coverage (%)	Shannon index	Simpson index	Chao1 index	observed_OTUs	Coverage (%)	Shannon index	Simpson index	Chao1 index
N6	2406.33 ± 394.96 ab	0.99 ± 0.01	10.15 ± 0.20a	0.9979 ± 0.0002ad	2564.09 ± 481.04 ab	920.67 ± 44.95a	1.00 ± 0.00	6.86 ± 0.44a	0.9619 ± 0.0196 ab	921.35 ± 45.43a
N7	2628.83 ± 143.70a	0.98 ± 0.00	10.26 ± 0.11 ab	0.9981 ± 0.0003 ab	2841.13 ± 167.34a	917.33 ± 109.76a	1.00 ± 0.00	6.61 ± 0.62a	0.9529 ± 0.0229a	920.61 ± 114.65a
N8	2529.00 ± 193.63 ab	0.99 ± 0.00	10.21 ± 0.17 ab	0.9982 ± 0.0002b	2726.10 ± 229.63 ab	742.17 ± 56.83b	1.00 ± 0.00	6.86 ± 0.14a	0.9734 ± 0.0055b	744.77 ± 61.20b
N9	2557.50 ± 244.37 ab	0.99 ± 0.00	10.31 ± 0.14b	0.9982 ± 0.0002b	2760.00 ± 318.38 ab	771.83 ± 37.61bd	1.00 ± 0.00	6.90 ± 0.34a	0.9702 ± 0.0104 ab	772.40 ± 37.54bd
N10	2316.67 ± 122.74b	0.99 ± 0.00	10.00 ± 0.05c	0.9976 ± 0.0002cd	2488.24 ± 161.12b	842.67 ± 69.47 acd	1.00 ± 0.00	6.95 ± 0.20a	0.9740 ± 0.0055b	843.21 ± 49.74cd

As shown in Table 2, the Chao1 index of bacterial community increased first and then decreased, with significant differences (*p* < 0.05), while the Chao1 index of fungal community showed a significant downward trend (*p* < 0.05), indicating that the richness of rhizosphere soil microbial community eventually decreased with the extension of planting time. The Shannon index and Simpson index of the bacterial community showed a significant difference (*p* < 0.05), while the Shannon index and Simpson index of the fungal community did not change significantly, indicating that the diversity of the bacterial community decreased with the extension of the planting time, while the fungi did not change significantly. Pearson correlation analysis (Figure 2C) showed that the α diversity index was affected by soil properties, among which Fe was significantly negatively correlated with fungal Shannon index (*p* < 0.001) and fungal Simpson index (*p* < 0.05), organic matter was negatively correlated with bacterial Shannon index and Simpson index (*p* < 0.05), and Prot was negatively correlated with fungal Chao1 index (*p* < 0.05).

At the genus level, there were 475 OTUs in soil bacteria (Figure 2D), and the least number of N10-specific OTUs (40), and the number of OTUs unique to different months decreased with the extension of planting time. There were 255 OTUs in soil fungi (Figure 2E), and the least number of OTUs endemic to N8 (22) was the same.

### 3.4 Distribution of soil bacterial and fungal community structure

**FIGURE 3:**
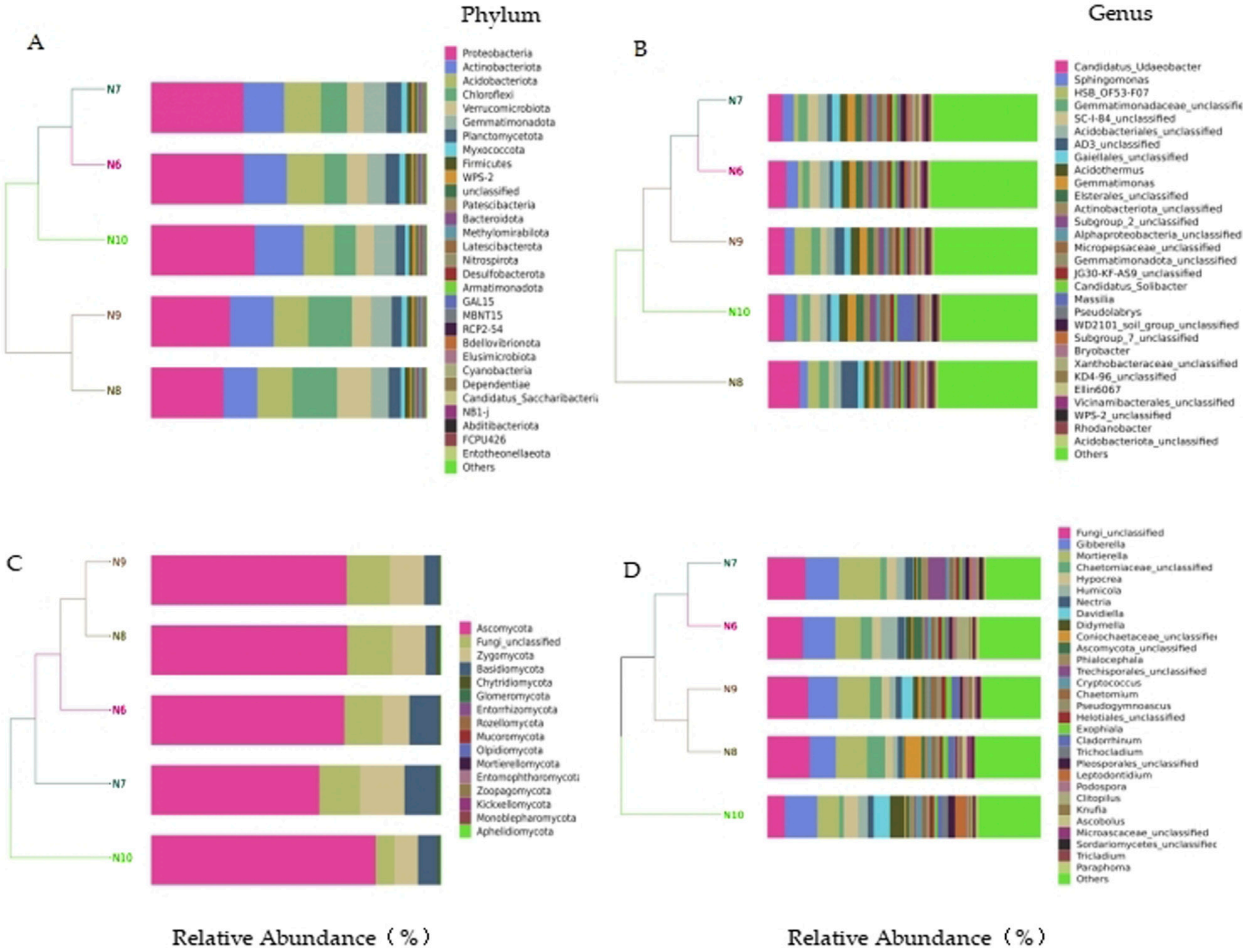
Species distribution of soil microbial communities. **(A)** bacterial phylum level; **(B)** bacterial level; **(C)** Fungal phylum level; **(D)** fungal level.

In the bacterial community, a total of 10 phyla with a relative abundance of more than 1% were identified, including Chloroflexi, Myxococcota, Proteobacteria, Actinobacteriota, Firmicutes, Acidobacteriota, WPS-2, and Blastomonas Gemmatimonadota), Planctomycetota, and Verrucomicrobiota. The top eight bacterial genera in relative abundance were Candidatus_Udaeobacter (7.24%), Sphingomonas (3.93%), HSB_OF53-F07 (3.25%), Gemmatimonadaceae_unclassified (3.13%), SC-I-84_unclassified (3.13%), Acidobacteriales_unclassified (3.09%), AD3_unclassified (2.84%) and Acidothermus (2.77%). In the fungal community, four phyla with relative abundance of more than 1% were identified, namely Ascomycota, Fungi_unclassified, Basidiomycota and Zygomycota. The top eight fungal genera in relative abundance were Fungi_unclassified (12.79%), Gibberella (11.29%), Mortierella (11.21%), Chaetomiaceae_unclassified (3.75%), Hypocrea (3.62%), Humicola (3.38%), Nectria (2.28%) and Davidiella (2.21%) (Figure 3 **)**.

The results of cluster analysis showed that the bacterial and fungal communities of N10 could not be clustered with other months, indicating that the soil microbial composition of N10 was quite different from that of other months.

### 3.5 Comparative analysis of soil bacterial and fungal communities

**FIGURE 4:**
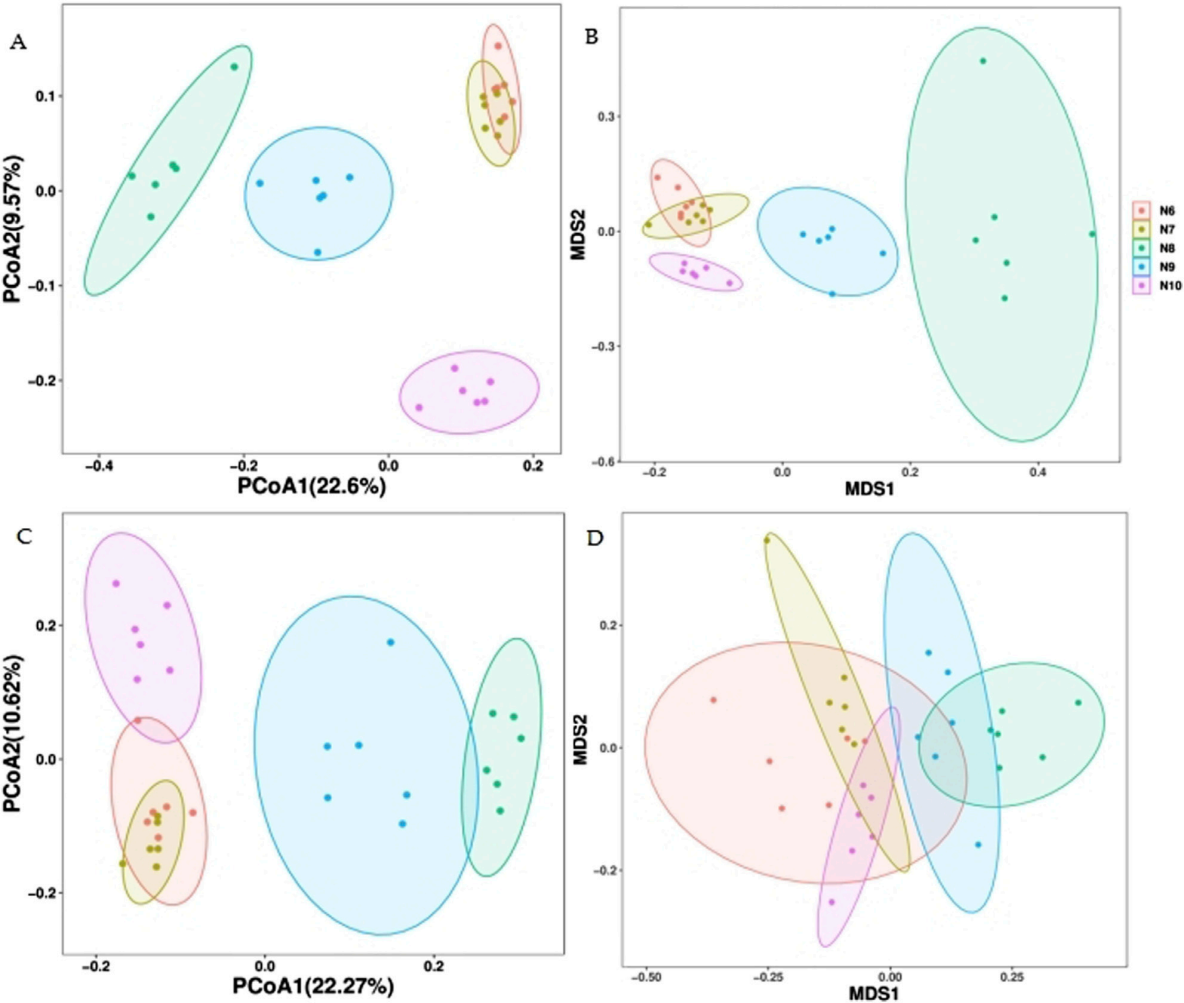
Primary coordinate analysis (PCOA) of soil microbial community β diversity at Bray-Curtis distance. **(A)** bacteria; **(C)** fungus and non-metric multidimensional scale (NMDS) **(B)** bacteria; **(D)** fungus.

As can be seen from the principal coordinate analysis (PCOA) plot of the Bray-Curtis distance (Figure 4), the N8, N9 and N10 soil bacterial communities can be well distinguished from the groups, but the soil bacterial communities of N6 and N7 cannot be well distinguished, as evidenced by the non-metric multidimensional scale (NMDS) of the Bray-Curtis distance. In the fungal community, the PCOA of the Bray-Curtis distance could distinguish N8 and N9 from N6, N7 and N10 well, but the NMDS could not distinguish the groups well, indicating that the soil fungal community was less different from the bacterial community in different months.

### 3.6 Functional gene analysis of soil microbial communities in the rhizosphere of ginseng

**FIGURE 5:**
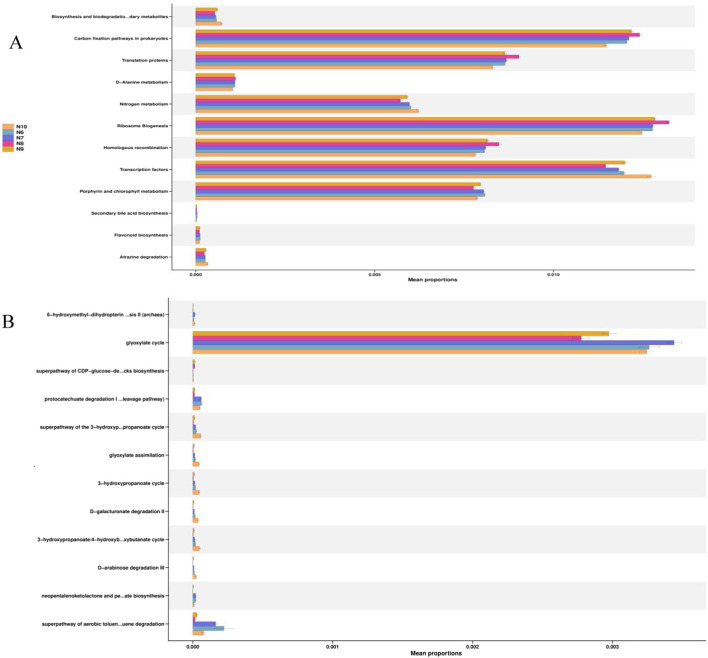
Relative abundance of KEGG pathway in the bacterial **(A)** and fungal **(B)** communities in the rhizosphere soil of ginseng at different stages.

**FIGURE 6:**
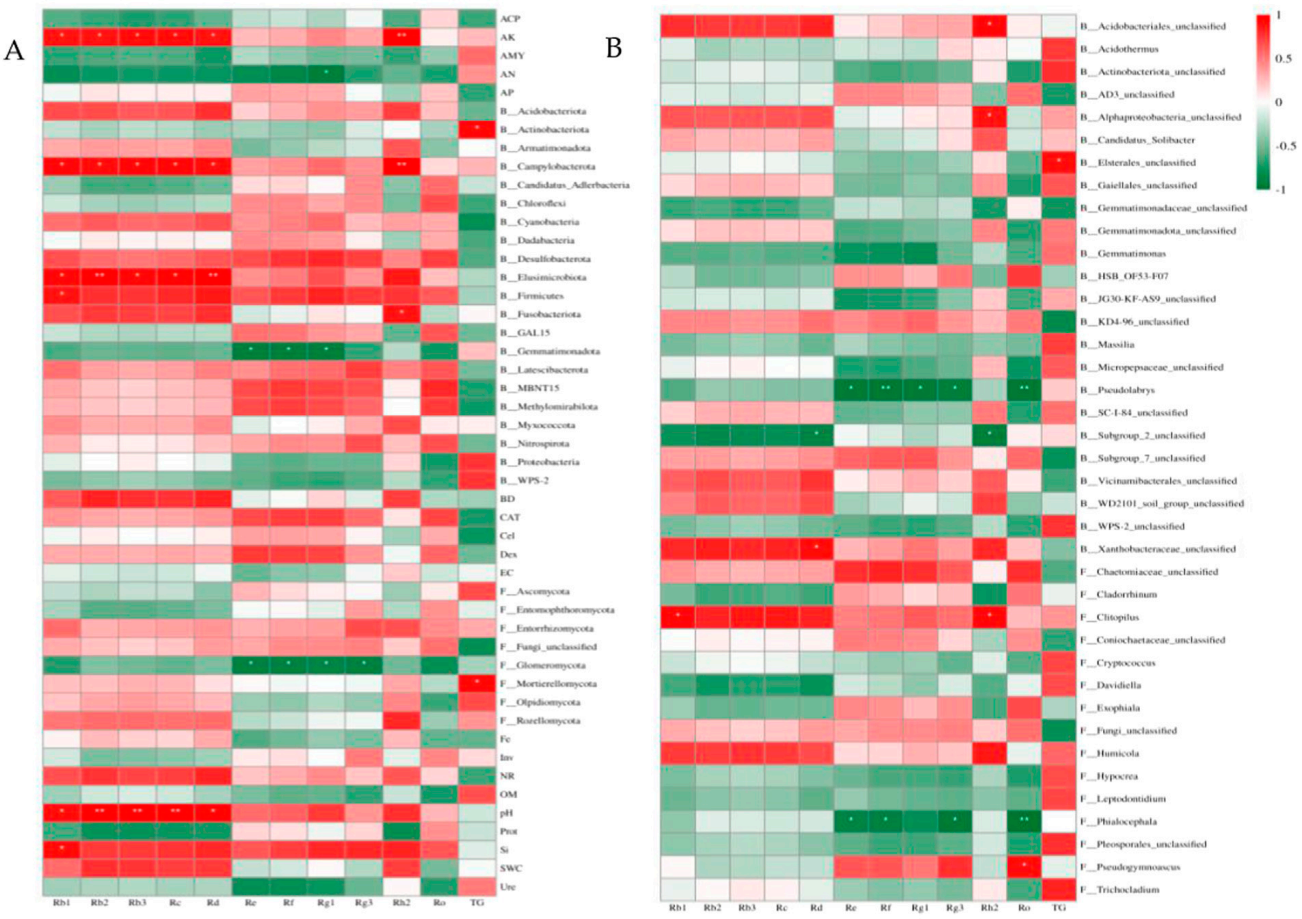
Pearson correlation analysis of ginsenosides with soil factors and soil microbial communities. **(A)** Correlation analysis of ginsenosides at the phylum level with soil factors, soil bacteria and fungal communities, **(B)** Correlation analysis between ginsenosides and soil bacterial and fungal communities at the genus level, B: bacteria, F: fungi.

In order to obtain the functions of soil bacteria and fungi, the KEGG database pathway level 3 was used to compare the relative abundance of functional genes of soil microbial communities, and the results are shown in Figure 5. It can be seen that carbon fixation, translational protein, nitrogen metabolism, ribosomal biogenesis, homologous recombination, transcription factors, porphyrin and chlorophyll metabolism in prokaryotes are the main functions of the ginseng rhizosphere soil bacterial community at different stages (Figure 6A). The glyoxylic acid cycle is the main function of the fungal community (Figure 6B).

### 3.7 Relationship between ginsenosides and soil factors and soil microbial communities

Correlation analysis and redundancy analysis (RDA) were used to identify soil factors and soil microbial communities that had significant effects on ginsenosides. The correlation analysis between microbial communities and ginsenosides at the phylum and genus levels with soil factors, relative abundance of >1% and significant differences between different months (*p* < 0.05) was analyzed, and the results were shown in Figure 6, AK, AN, pH, Si, Actinomycetes, Campylobacterota, Elusimicrobiota, Firmicutes, Blastomonas and Glomeromycota and Mortierellomycota were significantly correlated with ginsenosides (*p* < 0.05), Acidobacterium (Acidobacteriales_unclassified), α-Proteus (Alphaproteobacteria_unclassified), Elsterales_unclassified, Pseudolabrys, Subgroup_ 2_unclassified. There was a significant correlation between Flavobacter (Xanthobacteraceae_unclassified), Clitopilus, Phialocephala, and Pseudogymnoascus (*p* < 0.05).

**FIGURE 7:**
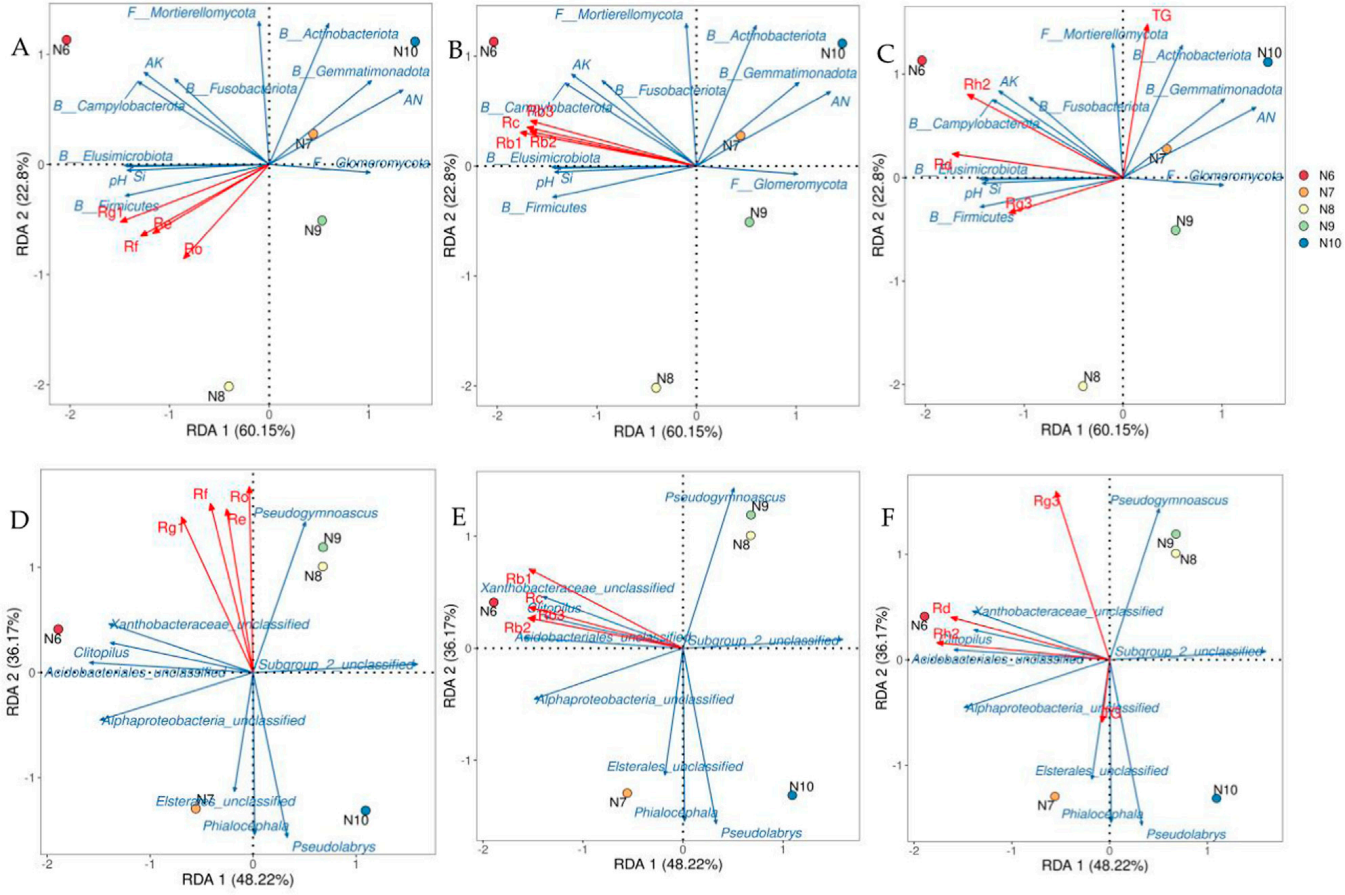
Redundant analysis of ginsenosides, soil factors, and soil microbial community structure. Note: **(A–C)** were redundant analyses of soil microbial communities at the level of ginsenosides and soil factors and phylums. Redundancy analysis of soil microbial communities at the level of ginsenosides and genus **(E–F)** B: bacteria; F: Fungi.

The soil factors and microbial communities which were significantly correlated with ginsenoside (*p* < 0.05) were screened by correlation analysis, and the redundancy analysis with ginsenoside was carried out. As shown in Figure 7, the interpretation rates of the first and second axes for soil properties and microbial communities were 22.80%, 60.15% (Figures 7A–C) and 36.17%, 48.22% (Figures 7D–F), respectively, the total explanation rate was 82.95% and 84.39%. Rb1 (*r* ^2^ = 0.9955, *p* = 0.0083) was significantly correlated with soil factors and microbial phylum (Figure 7B), and Rh2 was significantly correlated with soil factors and microbial phylum (*r* ^2^ = 0.9646, *p* = 0.049, Figure 7C) and genus (*r* ^2^ = 0.9699, *p* = 0.0167, Figure 7F). Combined with the results of Pearson correlation analysis, the significant positive correlation between Rb1 and Si, pH, AK, Firmicutes, Elusimicrobiota and Campylobacterota was consistent. Rh2 was significantly positively correlated with pH, AK, Fusobacteriota, Campylobacterota, Acidobacteriales_unclassified, Alphaproteobacteria_unclassified and Clitopilus, and the significant negative correlation with Subgroup_2_unclassified was supported by consistency.

## 4 Discussion

In this study, the contents of 11 monomeric saponins and total saponins in 3-year-old field ginseng from June to October were determined, and the physicochemical properties, enzyme activities and soil microbial communities of rhizosphere soil were measured in the corresponding period. Correlation analysis combined with redundancy analysis showed that Si, *pH*, AK, Firmicutes, Elusimicrobiota, Campylobacterota were significantly correlated with the accumulation of Rb1, *pH*, AK, Fusobacteriota, Campylobacterota, Acidobacteriales_unclassified, Alphaproteobacteria_ Unclassified, Clitopilus, and Subgroup_2_unclassified were significantly correlated with the accumulation of Rh2.

It was found that the soil *pH* value was less than five in the third year of *P. ginseng* cultivation, which was not in the range suitable for ginseng growth, because the soil was measured in the third year of ginseng planting, so it may be due to the soil acidification caused by ginseng planting, resulting in the soil pH not in the most suitable range for ginseng growth. Ginseng secretes phenolic acids into the soil during the growth process, and the soil acidity increases with the extension of the ginseng cultivation period, and it is generally believed that the soil *pH* of 5.5–6.5 is the most suitable range for ginseng growth (You et al., 2015; Wu et al., 2019; Xu et al., 2020). Ginsenosides Rb1 and Rh2 were still significantly positively correlated with *pH* (*p* < 0.05), which indicated that the *pH* range of 4.39–4.77 may contribute to the accumulation of two kinds of ginsenosides, Rb1 and Rh2. Rb1 was significantly positively correlated with AK, which was consistent with the results of Jie et al. (2017). The soil environment and growth of ginseng in farmland and under forest were summarized. The content of available potassium in soil suitable for ginseng cultivation was 200–300 mg/kg (Aixian et al., 2018). The available potassium content in this study was 17.78–139.76 mg/kg, which indicated that the lower available potassium content may still contribute to the accumulation of Rb1 and Rh2 ginsenosides. The content of available potassium in soil was an important factor affecting the accumulation of Rb1 and Rh2.

The important component of soil microbial system not only participates in the decomposition and transformation of organic matter and the formation of humus, but also can regulate and improve plant nutrient status and form specific symbiotic relationship with plants. The change of microbial community structure in ginseng rhizosphere soil, the decrease of beneficial microbial activity, the increase of pathogenic fungi and the deterioration of soil properties led to the occurrence of many diseases, it has a great negative effect on the quality and yield of ginseng (Jin et al., 2022). Firmicutes is an oligotrophic bacterium that is more suitable for surviving in extreme environments, and these bacteria can take nutrients from the atmosphere and convert them into carbon and nitrogen sources to maintain normal growth (Langille et al., 2013). Acidobacteriales_unclassified bacteria are involved in the iron cycle, the metabolism of single-carbon compounds, and the degradation of plant residues (Barns et al., 2007; Lu et al., 2010), and often live in a lower pH environment, and changes in pH can affect the activity of these bacteria (Peng et al., 2021). The research showed there was a significant correlation between *pH* and Firmicutes, Acidobacteria. Ginsenosides Rb1 and Rh2 had significant positive correlation with soil *pH*. Changes in *pH* may affect the activity of Firmicutes and Acidobacteria, there by affecting the accumulation of ginsenosides. Firmicutes and Acidobacter are the key micro-organisms affecting ginsenosides.

Iron is involved in numerous biological metabolic processes, such as nitrogen fixation, maintenance of cellular enzyme activity, photosynthesis and respiration. Iron plays an important role in fungal growth and is one of the essential elements for fungal growth, and iron deficiency can lead to slow fungal growth (Johnson, 2008; Silva et al., 2020); The availability of organic matter is a key factor affecting microbial community composition and biomass. Soil microbial diversity rich in organic matter is also abundant (Accoe et al., 2004). Correlation analysis showed that organic matter and Fe were significantly negatively correlated with the α diversity index of bacterial and fungal communities, respectively (*p* < 0.05), indicating that the contents of organic matter and complexed Fe in soil were not suitable for the growth of microbial communities, or could not be absorbed and utilized by microorganisms. This may be one of the factors that indirectly affect ginsenoside accumulation.

The results of a number of mathematical statistics showed that *pH*, AK, Si, Firmicutes, Elusimicrobiota, Campylobacterota, *Fusobacterium*, Acidobacteriales_unclassified, Alphaproteobacteria_unclassified, Clitopilus and Subgroup_2_ in soil Unclassified is the key factor affecting the accumulation of ginsenosides, and these factors can be focused on in future ginseng cultivation. To reveal the interaction mechanism between soil and quality of ginseng, and to provide theoretical reference for soil improvement of ginseng.

## 5 Conclusion

The biosynthesis of ginsenoside is influenced by both genetic and environmental factors. *pH*, available potassium, organic matter, complexed iron, Firmicutes and Acidobacteria were the important influencing factors of ginsenoside. The changes of soil physical and chemical properties (*pH*, available potassium, organic matter and complexed iron) affect the abundance of soil microorganisms (Firmicutes and Acidobacter), and soil microorganisms affect the accumulation of ginsenoside in ginseng. A co-regulatory network of Physicochemical Properties-Microbe-Ginsenoside was established.

## Data Availability

The data presented in the study are deposited in the NCBI repository, accession numbers PRJNA1153287, and PRJNA1153137.

## Abbreviations

EC: Electrical Conductivity; BD: Bulk Density; SWC; Soil water content; AN: Alkali-hydrolyzable nitrogen; AK: Available Potassium; AP: Available Phosphorus; OM: Organic Matter; CAT: Catalase; Prot: Protease; Dex: Dextranase; Amy: Amylase; Cel: Cellulase; NR: Nitrate Reductase.
